# Incorporation of Barium Ions into Biomaterials: Dangerous Liaison or Potential Revolution?

**DOI:** 10.3390/ma14195772

**Published:** 2021-10-02

**Authors:** Ilijana Kovrlija, Janis Locs, Dagnija Loca

**Affiliations:** 1 Rudolfs Cimdins Riga Biomaterials Innovation and Development Centre, Faculty of Materials Science and Applied Chemistry, Institute of General Chemical Engineering, Riga Technical University, Pulka 3, LV-1007 Riga, Latvia; ilijana.kovrlija@rtu.lv (I.K.); janis.locs@rtu.lv (J.L.); 2 Baltic Biomaterials Centre of Excellence, Headquarters at Riga Technical University, Kaļķu Street 1, LV-1658 Riga, Latvia

**Keywords:** barium, biomaterials, hydrogels, physiology, bone tissue regeneration, calcium phosphate

## Abstract

In the present manuscript, a brief overview on barium, its possible utilization, and the aftermath of its behavior in organisms has been presented. As a bivalent cation, barium has the potential to be used in a myriad of biochemical reactions. A number of studies have exhibited both the unwanted outcome barium displayed and the advantages of barium laden compounds, tested in in vitro and in vivo settings. The plethora of prospective manipulations covered the area of hydrogels and calcium phosphates, with an end goal of examining barium’s future in the tissue engineering. However, majority of data revert to the research conducted in the 20th century, without investigating the mechanisms of action using current state-of-the-art technology. Having this in mind, set of questions that are needed for possible future research arose. Can barium be used as a substitute for other biologically relevant divalent cations? Will the incorporation of barium ions hamper the execution of the essential processes in the organism? Most importantly, can the benefits outweigh the harm?

## 1. Introduction

**Figure 1:**
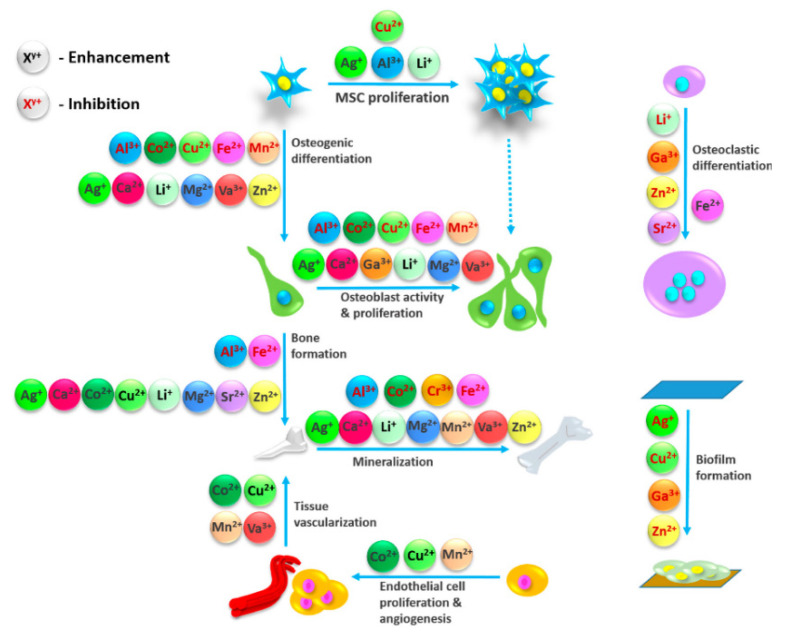
Effect of different ions in bone regeneration processes. Obtained from the reference [13].

Scaffolds used in bone tissue engineering have been under continuous scrutiny. Establishing an ideal construct that corresponds to the set goals of biocompatibility, biodegradability, promotion of bone regeneration, while at the same time mimicking the distinctive properties of natural bone, has proven to be strenuous [1]. One of the constituents that have taken the spotlight as the most promising, are calcium phosphates (CaPs). The spectrum of calcium phosphates encompasses twelve CaPs, with the Ca/P molar ratio ranging from 0.5 to 2.0 [2,3,4]. They are especially relevant due to the fact that they represent the inorganic part of bone and teeth [2], which is why they are closely mentioned with the process of biomineralization. “Biomineralization can be described as a phenomenon in which a mineral is integrated as a functional and often structural part of living organisms, often in direct and close contact to a matrix forming protein or carbohydrate structure” [5]. As a part of the bone, apatite is presumably formed from the non-stoichiometric and ion-doped CaPs, originating from amorphous form [2,6,7,8]. In a detailed in situ investigation, Habraken et al. [8] described the process as starting from the generated pre-nucleation complexes, called Posner’s clusters, which essentially are calcium triphosphate ion-association complexes. The next stage includes the nucleation of amorphous calcium phosphate (ACP), with a subsequent conversion to octacalcium phosphate (OCP), through a continuous binding of calcium ions (Ca^2+^). The postulated mechanism ends with the formation of apatite, embodying the calcium triphosphate complex as its fundamental structural unit. The well-established crystal structure of CaPs, included within the ternary system Ca(OH)_2_–H_3_PO_4_–H_2_O, enables the transitions from one form to another (e.g., layer-by-layer growth mechanism of HAp through OCP), as well as numerous incorporations [9]. Functionalization of CaPs with miscellaneous ions has proven to be beneficial in diverse stages of bone regeneration processes (Figure 1). Up until now, multiple ions have been used to steer the pathways of complex mechanisms transpiring in the body. These ions range from vanadium (V^5+^), niobium (Nb^5+^), boron (B^3+^), gallium (Ga^3+^), and iron (Fe^3+^) to calcium (Ca^2+^), cobalt (Co^2+^), copper(II) (Cu^2+^), magnesium (Mg^2+^), strontium (Sr^2+^), zinc (Zn^2+^), lithium (Li^+^), silver (Ag^+^), fluoride (F^−^), bromide (Br^−^), chloride (Cl^−^), hydroxyl (OH^−^), hydrogen phosphate (HPO_4_^2−^), carbonate (CO_3_^2−^), phosphate (PO_4_^3−^), and silicate (Si^4−^) [10,11,12,13,14,15,16,17]. Cationic substitutions of CaP, e.g., HAp generalized through a formula M_10_(XO_4_)_6_Y_2_, where M is typically a bivalent cation, can occur with a complete or partial replacement of Ca^2+^ [16]. Depending on an ionic radius and concentration, these substitutions can either stabilize the structure or destabilize the lattice [18,19].

Furthermore, ions have not only been used to ameliorate the structure, morphology, and the effect CaPs have, but they have been used also as crosslinking agents for hydrogels [20]. Hydrogels represent a three-dimensional hydrophilic polymer network with high affinity to water. Depending on their crosslinking approach (chemical or physical), properties such as reduced dissolution or distinctive mechanical and biochemical properties with various functions (e.g., incorporation of 0.61 wt.% of Zn^2+^ in HAp resulted in the extension of their lag time, increasing its antibacterial potential) can materialize [21]. One of the physical approaches of hydrogel crosslinking is with ionic/electrostatic interactions (more information in Section 3.1) [21]. Even though many studies have examined the influence of bivalent cations such as Mg^2+^ [22], Sr^2+^ [23], and Zn^2+^ [24], there is a scarcity of information regarding the effect of one more alkaline earth metal—barium (Ba^2+^). Barium’s participation in bone repair and regeneration has placed it at the forefront of recent interest. In nature, barium does not occur in its free ionic form, but as a number of natural salt compounds, e.g., barite (BaSO_4_) or barium chloride (BaCl_2_) [25]. Barium compounds that are relatively soluble in water are acetate, nitrate, and halides (except fluoride), while compounds with carbonate, chromate, fluoride, oxalate, phosphate, and sulfate are fairly insoluble in water (Table 1) [26]. Bearing the benefits of the ion incorporation in mind, a question arises—why has the influence of barium on CaPs and their composites not been examined more in depth?

## 2. Barium within the Human and Animal Body

Numerous different ions can be found throughout the human body (e.g., sodium (Na^+^) [27,28], potassium (K^+^) [29,30], calcium (Ca^2+^) [31,32], magnesium (Mg^2+^) [33,34], strontium (Sr^2+^) [35,36], copper (Cu^2+^) [37,38], zinc (Zn^2+^) [38,39], iron (Fe^3+^) [40,41]), presenting the essential part of various mechanisms and processes that govern the functioning ability of an entire organism [17,42,43].

Barium is introduced to the human body usually via ingestion—mostly food (Brazil nuts, seaweed, fish) and water, inhalation, or skin contact [25,26,44]. According to the literature, Ba^2+^ content within the body does not increase regularly with age [44], and moreover, the average recorded concentrations of barium can vary depending on the region and the way of life [45,46]. Infants have exhibited the intake of 7.0 ± 4.0 ppm, while adults have slightly higher dose, 8.5 ± 4 ppm [47]. Even though it is not perceived as a bio-essential element, it has been revealed that the absorbed barium ions are distributed via the blood stream and deposited primarily in the bones (roughly 90% of the body burden, ranging from 0.5–10 µg/g [25]) [48,49,50]. In addition, Ba^2+^ has also been detected in teeth (0.1–3 µg/g), heart, blood, lung, pancreas, kidney, eyes, liver, aorta, brain, eyes, skeletal muscles, spleen, placenta, hair, and urine [25]. Notwithstanding the fact that Ba^2+^ is mostly accumulated in the skeleton, few data exist on the mechanism by which Ba^2+^ is deposited. Having a larger ionic radii than Ca^2+^ (1.34 Å, hexacoordinated to six negatively charged oxygen atoms, in contrast to 0.99 Å for Ca^2+^ [49,51]), Ba^2+^ has a greater possibility to be eliminated in the process of recrystallization of hydroxyapatite (HAp) [49]. Due to this, it is perceivable to assume another mechanism that is taking place in the deposition of Ba^2+^ in the bone tissue. In the opinion of Schubert and Conn (1949) and Jowsey, Rowland, and Marshall (1958), described in the paper of Bligh and Taylor [49], the reaction between barium ions and phosphorous ions (PO_4_^3−^) can be associated with the behavior barium has with sulfate ions (SO_4_^2−^). While forming barium sulfate, a radiocolloid is being formed, despite the fact that the overall concentration of the ions is insufficient to exceed the normal solubility product of the compound [49]. Given this, it could be possible that barium is adsorbed on the surface of bone structural elements in the form of colloidal particles due to the reaction with PO_4_^3−^. This process is thought to be not only restricted to the areas that are actively calcifying by ionic exchange with Ca^2+^, but also by the expeditious irreversible process of surface adsorption [49]. In order for Ba^2+^ to be predisposed for in vivo delivery to bone, it has to be in water-soluble form. Panahifar et al. [52] examined the spatial distribution of Ba^2+^ in the skeleton. The study showed that Ba^2+^ was principally integrated in mineralizing areas particularly in the growth plates of rats’ long bones (areas of cartilage located near the ends of bones [53]). The effect of age on Ba^2+^ uptake (dosage was 58.5 mg/kg/day, i.e., 33 mg/kg/day of free Ba^2+^) showed that young rats (one month old) incorporated 2.3-fold more Ba^2+^ in their bone than old rats (eight month old). Furthermore, Ba^2+^ was found in the endosteal and periosteal layers of cortical bone, as well as on the trabecular surfaces of epiphyses, suggesting appositional growth [48,52]. Compared to Sr^2+^, Ba^2+^ exhibited faster absorption from the gastrointestinal tract and faster incorporation in bone, but at a smaller concentration [48]. Studies comparing the effects of high-dose exposures and chronic low-dose exposures of barium on human health are in deficit. However, several records collected from animals claim that high uptake levels of Ba^2+^ (150–450 mg/kg/day) are connected with high blood pressure; kidney and liver failure; stimulation of smooth, striated, and cardiac muscles; and disorders of central nervous systems [25]. Reliable data on the shortage of barium in biological systems are scarce and do not contain the complete aftermath.

## 3. Barium Comprising Biomaterials and Their Biological Performance

Despite the fact that barium, as a divalent cation, has vast potential to be utilized in combination with biologically relevant biomaterials, the mechanism of apposition or the outcome of possible effects is insufficiently researched. Detailed review of literature has pointed out that when Ba^2+^ is combined with pertinent polymers (e.g., alginate or hyaluronic acid), it has promising results. Considerably fewer studies have underlined the ramifications of the Ba^2+^–CaP fusion. Nevertheless, positive data regarding mechanical properties and biocompatibility have been presented.

### 3.1. Barium Loaded Hydrogels

Hydrogels are hydrophilic, polymer-based systems that absorb and preserve large amounts of water [54,55]. When making hydrogels, a sort of a crosslink is formed, whether through chemical crosslinking (covalent or ionic bonds) or physical crosslinking (ionic forces or electrostatic forces). In addition, van der Waals forces and hydrogen bonds can also operate as crosslinks [56].

**Figure 2:**
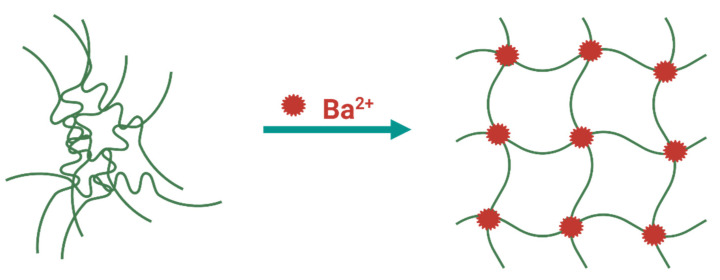
Schematic representation of barium crosslinking. Figure created with BioRender.com.

One of the ways physically crosslinked hydrogels can be synthesized is through the interplay of various ions at mild conditions (room temperature and physiological pH). A hydrogel with stronger properties will be achieved by using metallic ions due to the coordination stemming from Lewis acid–base interactions [54,55]. Commonly, the most explored hydrogels, crosslinked with metal ions, are those with coordination tethered by metal cations [57]. For this purpose, cations such as Fe^3+^, Ca^2+^, Sr^2+^, and Zn^2+^ are widely used. Barium ions have the ability to form salts with particularly low solubility in aqueous media. On this accord, several studies [58,59,60,61,62,63] have examined the effect of barium crosslinking on the overall properties of different polymers. As a divalent cation, Ba^2+^ usually forms ionic crosslinks, which transpire as a prerequisite of achieving electrical neutrality in the material (Figure 2) [58]. Barium has the capability of establishing two crosslinking mechanisms within the materials, already mentioned ionic crosslinks, and physical crosslinks [58]. Ionic crosslinks are independent of temperature, while physically crosslinked materials are supposed to be temperature-dependent. Further distinction between these two mechanisms is that physically crosslinked materials are formed owing to ion–dipole associations of the BaSO_4_ groups, producing ionic aggregation, i.e., ion clusters. Ion clusters secure versatile crosslinks constructed by nano-phase separation of ion-rich domains (1–5 nm). In order to prove which type of crosslinking has transpired, structural analysis is required.

**Figure 3:**
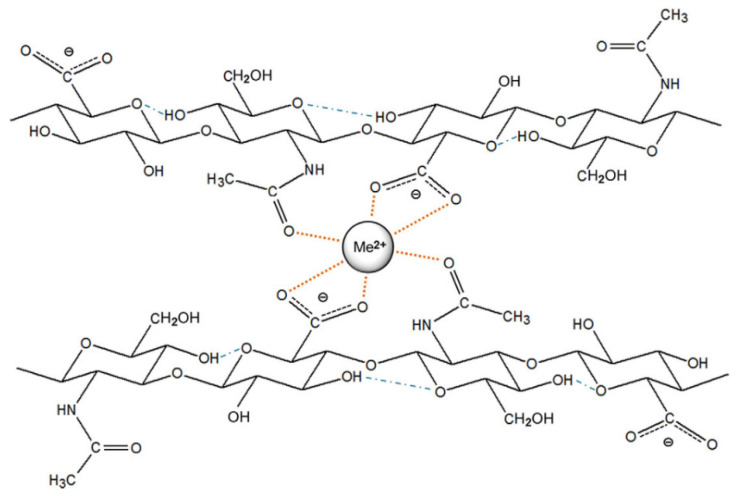
Formed chelate structure between a bivalent cation and two disaccharide units. Reprinted with permission from ref. [59]. Copyright 2013 Elsevier.

In the study conducted by Gasa et al., barium was used on acidic polymer electrolyte membranes (PEM), based on sulfonated poly(ether ketone ketone) (SPEKK), so as to reduce the sorption of aqueous media and improve their mechanical properties and stability [58]. The crosslinking between sulfonate groups occurred by the exchange of barium ions with the protons in SPEKK membranes. The increase in the exchanged barium resulted in the decrease in equilibrium water sorption (17 wt.%). However, when the Ba^2+^ exchange was above 64%, the fluid uptake was practically independent of temperature and methanol activity in water–ethanol solutions. Nonetheless, if the percentage of exchanged cation was lower, the temperature dependence was visible (<45 °C weak dependence, >45 °C sharp upturn in the water sorption). The reason for this behavior is most likely the glass-to-rubbery state transition of the water-swollen SPEKK. Moreover, thermal stability was considerably improved in dry conditions. As was mentioned before, the size of the barium ions is substantial in comparison to others; hence, they exhibit less mobility than the mobile protons that were interchanged. When combined with the partially deprotonated hyaluronic acid, barium (similar to other bivalent cations) results in the formation of chelate-like complexes (Figure 3), followed by an increasing degree of cross-linking within or between polymer chains [59]. The viscosity of the hyaluronate solution was substantially lowered with an increase in cation concentration, while the conformation was radically changed.

In a method for the microencapsulation of sensitive drugs (bovine serum albumin (BSA)) within the carboxymethyl guar gum (CMGG), Thimma and Tammishetti [61] investigated the benefits of crosslinking the polymer with Ba^2+^ instead of Ca^2+^. On account of the preformed swelling studies, barium crosslinking was more efficient at all concentrations that were tried, the reason probably being the larger ionic radii, which brings two different carboxylate ions closer in respect of the conformation.

**Figure 4:**
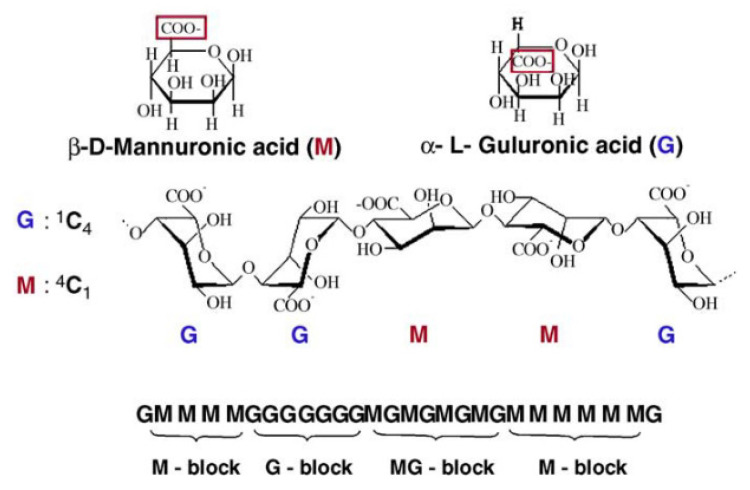
The structure of linear block co-polymer alginate comprising β-D-mannuronic (M) and α-L-guluronic acids (G). Reprinted with permission from ref. [64]. Copyright 2006 Elsevier.

Conversely, the majority of papers were associated with crosslinking of barium and alginate [60,62,64,65,66,67,68]. Alginate is a linear block co-polymer, comprising β-D-mannuronic (M) and α-L-guluronic acids (G) (Figure 4). Arrangements of M and G blocks can have numerous variations [64]. Barium forms stronger bonds with alginate gels than calcium for both GG blocks and MM blocks [69]. Nonetheless, alginates that possess a higher G block content (more than 60% G) are endowed with stronger bonds, whereas the stability enforcement is missing for alginates augmented with more M blocks (less than 40% G) [70].

**Figure 5:**
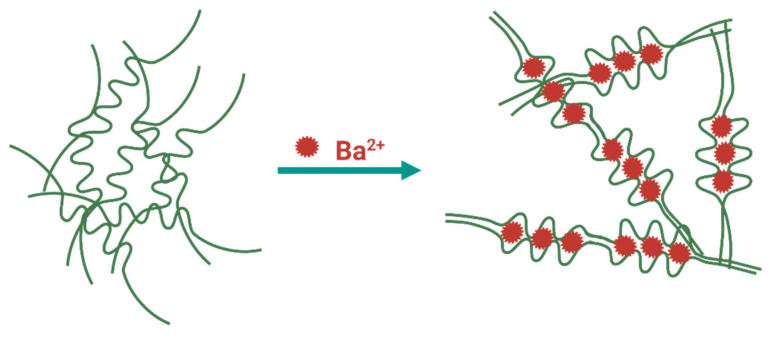
Schematic representation of ion crosslinking of alginate gel by the “egg box” model. Figure created with BioRender.com.

The “egg box model” is commonly used to describe the formation of alginate gels (Figure 5). The divalent ions interacted jointly with G blocks to form ionic bridges between adjoining chains. The reactivity and gel formation capacity were directly correlated with the average chain length of the G blocks [62,71].

Due to this specific binding and the size of the ion itself, the barium crosslinked gel manifested a lower swelling degree; thus, it was more stable in aqueous media [62]. In a study by Bajpai et al. [66,72], alginate-formed beads were placed in a buffer medium with pH 7.4. Barium ions bounded to the carboxylic (COO^−^) groups, starting the process of exchange with sodium ions situated in the swelling medium. After the maximum swelling of the beads was achieved, barium ions in the egg box junctions started to diffuse out and the beads began slowly disintegrating over a longer period of time, owing to the ion size [66]. Some of the effects ascribed to the barium crosslinking are summarized in Table 2.

Based on the thorough read-through of the papers summarized in Table 2, the lack of research on biological outcomes is evident. The paucity of information regarding the association of barium and different types of polymers and their effect on biological performance can be credited to only several papers, dating back to the 1990s [73,74,75]. The focus of their work was based on microencapsulation of rat islets with barium chloride (BaCl_2_) crosslinked alginate. The capsules were biocompatible for syngeneic and allogeneic transplanted islets in diabetic BALB/c and non-obese diabetic (NOD) mice. They demonstrated that normoglycemia was attained in all STZ-induced diabetic NOD mice transplanted with islets encapsulated in the barium–alginate complex. Furthermore, islets were able to reverse diabetes for almost a year, proving that the dynamics of insulin release from microcapsules are fast enough [73]. Gröhn et al. [75] examined the growth of anchorage-dependent cells (human Chang liver (CCL-13) and mouse fibroblast (L929) cell lines) and they observed that after 24 h, the cells grew rapidly and reached confluence after three days on the barium crosslinked matrix. However, even with the promising results several of these groups obtained, no detailed work on further barium use was performed.

### 3.2. Synthesis of Calcium Phosphates Containing Barium

As a divalent cation, barium extends the possibility of being incorporated within different calcium phosphates. There have been few studies concerning the preparation of barium–calcium apatites [76,77,78,79,80]. Bigi et al. tried to form a barium–calcium hydroxyapatite (BaCaHAp) by a solid state reaction at 1200 °C and by a precipitation method at 100 °C [81]. The products obtained by the solid state reaction, at high temperatures, covered the array of barium concentrations from 0 to 100 atom%. By using that method, lattice dimensions and the FT-IR absorption frequencies displayed a linear increase, following the increase in the atom% of Ba^2+^. Only small quantities of Ba^2+^ were incorporated in HAp by precipitation from the aqueous system. Liu et al. synthesized calcium phosphate cement (CPC) powder with a mixture of α-tricalcium phosphate (α-TCP) and dicalcium phosphate dihydrate (DCPD) at the mass ratio of 9:1, with the addition of 20 wt.% starch and 20 wt.% BaSO_4_ [79]. Their aim was to look into the effects of BaSO_4_ on injectability and radiopacity, as well as the mechanical and biocompatibility properties of the CPC system. The compressive strength of the construct increased to over 50 MPa, with the injectability index higher than 90% (50 N at a constant injection speed of approximately 10 mm/min). In addition, the recorded radiopacity was high, while the setting times and biodegradation behavior was satisfying. Moreover, in vitro tests on hemolysis, endotoxins, and apoptosis, as well as subcutaneous implantation in vivo, demonstrated that the barium-laden cement was nontoxic and biocompatible. In another example of doping α-TCP with Ba^2+^ [82], stoichiometric amounts of ammonium dihydrogen phosphate (NH_4_H_2_PO_4_) and barium carbonate (BaCO_3_) were used with an end product of Ba-substituted α-TCP, (Ca_1-x_Ba_x_)_3_(PO_4_)_2_ (x = 0.05, 0.10, and 0.15). The results showed that the unit-cell volumes of the product were larger than that of undoped product (undoped a = 12.87271 Å, b = 27.28034 Å, c = 15.21275 Å; doped a = 13.0965 Å, b = 27.9046 Å, c = 15.4021 Å), which would suggest that the reactivity of barium-doped α-TCP is higher. Yasukawa and his team synthesized carbonated BaCaHAp solid solution, with different Ba/(Ba + Ca) (XBa) atomic ratios (0–1) using the wet precipitation method at 100 °C [78]. Their results showed that no pure BaCaHAp was able to form, due to the irreversible adsorption amount of carbon dioxide (CO_2_). However, it should be noted that the information on the substitution efficiency of barium was not mentioned in the study. Yoder et al. synthesized carbonated barium hydroxylapatite (CBaApOH) and carbonated barium chlorapatite (CBaApCl) by aqueous synthesis. The end goal was to define the mechanism of carbonate substitution at 60 or 90 °C, as before, it was only preformed at solid-state, high-temperature synthesis [77]. Their main conclusions were that the synthesis parameters had to be closely monitored to avoid the precipitation of simple salts (BaCO_3_, Ba_3_(PO_4_)_2_, and BaApCl), mainly because of their close molar solubilities. CBaApCl and CBaApOH demonstrated solubilities that are marginally higher than the solubilities of their noncarbonated analogs, at low carbonate concentrations.

## 4. Biological Influence of Barium

Several studies have reported that barium-laden materials provide a favorable environment for cells and array of divergent functions [68]. The actual data collected on the overall biological influence of barium, incorporated in various calcium phosphates and hydrogels, are still scarce. With respect to the possible use of barium, instead of other divalent cations, Sarker et al. examined cell survival over time by assessing Schwann cell viability in the double-layered alginate strands [20]. In comparison to calcium, alginate strands crosslinked with BaCl_2_ (the concentration of alginate precursor was 2% and 3%, and Ba^2+^ = 50 mM) had intermediate values of Schwann cell viability (around 63%). However, when alginate microbeads, crosslinked with barium ion, were used to encapsulate Sertoli cells, excellent cell viability (90%) was noted after nine days of encapsulation (1% BaCl_2_ gelling solution) [65]. In addition, cells inside the beads were viable and formed tubule-like structures, while capsules had no loss of their functional and morphological properties for 8 months after transplantation. Myat-Htun et al. delved into in vitro bioactivity of barium-doped akermanite ceramic [83]. Akermanite powders were synthesized with calcium oxide, magnesium oxide, and barium oxide, with different Ba^2+^ contents (1, 3, and 5 mol%). An increase in the barium content caused a minimal shift in the X-ray diffraction peaks towards the smaller angles, while the crystallite size decreased (control sample 53.98 nm, 51.16, 49.45, and 49.36 nm for 1, 3, and 5 mol%, respectively). Relative density increased substantially when barium was introduced in the system (control 62.67 ± 0.27 and 5 mol% Ba 94.25 ± 0.12). The reason behind this effect is that Ba^2+^ is a sintering additive, introducing the formation of liquid-phase sintering and densifying the akermanite. Moreover, densification of the doped akermanite, with an increasing Ba^2+^ concentration, was observed (0.86 ± 0.01 GPa to 5.06 ± 0.14 GPa). The increase in Ba^2+^ content substantially increased the ability to form apatite (following 21-day SBF immersion, substrate peaks declined, and the new phosphate peaks of HAp were formed). Hence, the results of Fourier transform infrared spectrometry (FT-IR) confirmed in vitro growth of bone-like apatite, with an enhancement in growth ability, and no negative influences on chemical stability. Bioactive glass substituted with barium showed similar results [84]. In vitro tests were performed by immersing barium intercalated glasses in SBF media for 1, 3, 7, 14, and 30 days. The formation of a hydroxy carbonate apatite layer (HCA) transpired, and it was confirmed using FTIR and X-ray diffraction (XRD). Moreover, hemolysis assays displayed that biocompatibility improved in all the bioactive glasses on account of the barium oxide content (Ba-0 = 8.7%, Ba-1 = 6.5%, Ba-2 = 4.2%, Ba-3 = 3.1%, and Ba-4 = 5.4%, for Ba 0, 0.4, 0.8, 1.2, and 1.6 mol%, respectively). Hemolysis is the breakage of the red blood cell (RBC) membrane, causing the release of hemoglobin, directly correlated with biocompatibility. Acid citrate dextrose (ACD) human blood was used for these analyses. Acarturk et al. studied the impact of barium sulfate on remodeling and regeneration in standard tibial defects in rabbits treated with the Norian skeletal repair system (SRS) [85]. The SRS cement with barium (control—SRS cement without barium) manifested signs of biocompatibility and osteoconductivity after 6 weeks, while at the same time, it showed no evidence of inflammation of fibrous tissue around the implant materials or at the bone–implant material interfaces. Furthermore, even after a 2-year observation period, from a bone healing standpoint, the addition of barium had no negative effect on the osteophilic properties of SRS cement [85]. When barium was used as a crosslinking agent in alginate microcapsules, with stem cells of fibroblasts and U937 cells (a human cell line established from a diffuse histiocytic lymphoma), it manifested a proliferation of cells of 21 ± 2 fold and 6.6 ± 0.6 fold after 7 days, respectively. In addition, the same system showed that the encapsulated osteoblast cells could proliferate significantly and deposit calcium and alkaline phosphatase (ALP), reaching 13.5 ± 1.5 fold after 21 days [67].

Three independent studies have also underlined the role of barium incorporation as an ameliorating component for drug delivery systems [61,72,86]. Barium was used in combination with carboxymethyl guar gum and alginate. The formed system was investigated to be used for oral drug delivery along the gastrointestinal tract. The release of the drug (vitamin B12) was nearly 20% in the simulating gastric fluid (SGF) within the first 3 h, while 70% of drug was released in the next 7 h in the simulating intestinal fluid of pH 7.4 [86]. As a second drug, BSA was encapsulated and its release in vitro, in simulated gastric (after 1 day, a third of the total drug escaped) and intestinal buffers (80% of the BSA encapsulated was released in 4 h), was investigated [61]. Retention studies and loading efficiency was tested, and 53% of BSA was retained in the beads using a 1.25 M BaCl_2_ solution, while maximum loading was achieved using a solution containing 0.8% BSA.

## 5. Barium Toxicity

Even with a high potential of being a good substitute for commonly used metallic ions, barium has certain downsides. Knowing the current data available, and the fact that barium is known to be toxic, using barium as a crosslinking agent is still approached with caution [26,44,50]. Individuals’ sensitivity to barium toxicity as well as its role in epigenetic factors are correlated with specific geographic/geological areas, and the distributed information is quite limited [25]. In order to avoid these ramifications, extensive studies with different approaches are needed. For example, studies on the leakage of alginate gels crosslinked with Ba^2+^ ions have shown that when using low concentrations and exercising vigorous rinsing of barium beads, there is no leakage of the ion, and hence, no repercussions [64,87].

Although the data on the outcome barium has in in vivo settings are limited, a number of studies have delved more in to it. As Gallant [88] hypothesized early on, in 1982, the mechanism through which barium prompts negative effects is based on blocking the potassium channels in the cell membrane and promoting its transfer from an extracellular to intracellular media. In that study, Ba^2+^ emitted hypokalemic periodic paralysis of mammalian skeletal muscle and lowered the potassium ion serum concentration. Muscle bundles from Swiss–Webster mice and from pigs were accordingly prepared and deposited in the composition of solutions with 1–2 mM-Ba^2+^. The addition of Ba^2+^ resulted in blocking K^+^ channels in the membrane surface by entering them, causing the decrease in K^+^ conductance of neurons [88]. Walz et al. [89] reported similar conclusions while testing the effect of different concentrations of Ba^2+^ on the transport of potassium. Barium inhibited the unidirectional potassium influx (5 mM), the ouabain-sensitive net potassium uptake (IC_50_ of 0.6 mM), and Na^+^/K^+^-ATPase, which occurred with an IC_5O_ of 3.1 mM. IC_50_ is a half-maximal inhibitory concentration, which measures the potency of a substance in inhibiting a specific biological function.

Additionally, a major musculoskeletal effect detected in cases of barium toxicity in humans is progressive muscle weakness, often leading to partial or total paralysis [90]. Furthermore, Mores et al. monitored the effects of barium nanoparticles (1 g/L, 1 μg/L, 10 ng/L, and 1 ng/L) on the mononuclear (MN) cells of colostrum, which have an effect on a developing baby’s immune system [90]. The results they presented stated that barium lowered mononuclear phagocyte viability, heightened superoxide release, and reduced intracellular calcium release. Moreover, barium increased the cell death by apoptosis.

## 6. Future Directions and Conclusions

Despite being one of the metallic ions with all the characteristics ascribed to them (ionic radii, solubility, oxidation number, etc.), barium has not received comprehensive and diverse research. Most of the findings and knowhow on barium have their origin in the second half of the 20th century, with scarce follow-up in recent years.

**Figure 6:**
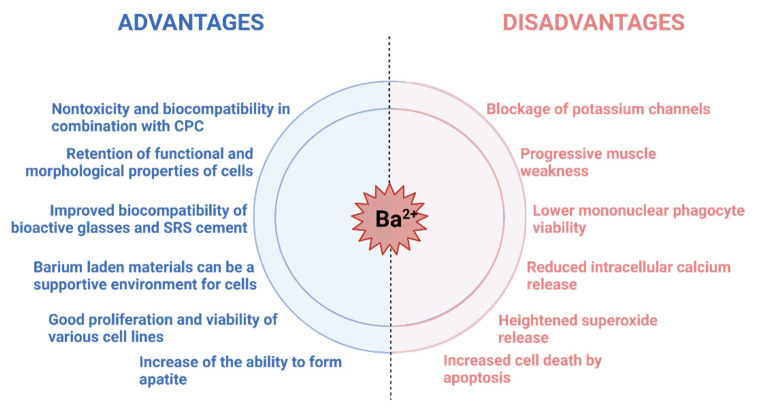
Brief overview of effects recorded in the presence of barium ions in vitro and in vivo. Figure created with BioRender.com.

In the previous sections, we presented a brief outlook on barium itself, its integration within hydrogels, and its potential to be merged with biologically relevant calcium phosphates (Figure 6). Biological influence and toxicity assessments have put barium in the shadow of the other important ions such as calcium, strontium, and zinc. Several sources have underlined the negative side of barium in organisms, including potential toxicity, blocking of potassium channels, lowering of cell viability, etc. However, the positive results should not be side-lined. The crosslinking of barium and polymers resulted in stronger matrix, lower swelling degree, tighter formation, and higher water resistance. As for the biological ramifications, islets embedded in microcapsules containing barium were able to reverse diabetes for almost a year. Furthermore, the cell lines CCL-13 and L929 grew rapidly and reached confluence after three days on a barium crosslinked matrix. Once barium was combined with calcium phosphates, such as HAp and α-TCP, the obtained cements exhibited nontoxicity and biocompatibility, with faster setting time. Moreover, in a separate study using akermanite as a starting point, the increase in the Ba^2+^ ratio increased the ability to form apatite.

Bearing all the information in mind, it must be emphasized that the toxic effects were for oral or intravenous administration of barium containing matrices. Consequently, the limited research on local barium’s influence on cells or antimicrobial properties should be further explored, as the shown potential and possibly much lower administrated dose rate are important factors. An added conclusion stemming from our thorough literature search is that the studies performed on barium incorporation, its effects, and influence are outdated. A fresh outlook on the overall behavior of barium and barium-loaded compounds is of vital importance. Use of state-of-the-art equipment and newly established methodologies will yield new discoveries and help to clarify the potential benefits that barium has to offer in the field of bone tissue regeneration and possibly propel barium to the forefront of tissue engineering.

## Figures and Tables

**Table 1:** Properties of barium and barium compounds [26].

Characteristics	Barium	Barium Acetate	Barium Carbonate	Barium Chloride	Barium Hydroxide	Barium Oxide	Barium Sulfate
Molecular formula	Ba	Ba(C_2_H_3_O_2_)_2_	BaCO_3_	BaCl_2_	Ba(OH)_2_x8H_2_O	BaO	BaSO_4_
Molecular weight	137.34	255.43	197.35	208.25	315.48	153.34	233.4
Melting point, °C	725	41	1740 (α form, at 90 atm)	963	78	1923	1580
Boiling point, °C	1640	No data	Decomposes	1560	550	2000	1149
Water solubility	Forms barium hydroxide	No data	0.02 at 20 °C, 0.06 at 100 °C	375 at 20 °C	56 at 20 °C, 947 at 78 °C	38 at 20 °C, 908 at 100 °C	0.00222 at 0 °C, 0.00413 at 100 °C
Specific gravity	3.5 at 20 °C	No data	4.43	3.856 at 24 °C	2.18 at 16 °C	5.72	4.5 at 15 °C

**Table 2:** Effects of Ba^2+^ cross-linked alginate on swelling and thermal stability, as well as on mechanical stability of hydrogels.

Biomaterial	Exhibited Effect	
	Swelling and Thermal Stability	Mechanical Stability
Alginate barium beads (600 kD) [64]	No data	Stability of alginate beads increased by replacing calcium for barium. With low concentrations and intensive rinsing of barium beads, no barium leakage was observed
Sodium-Alginate-based hydrogels [60]	Swelling degree (ϕ) (13–19% in deionized water and 12–17% in NaCl 0.15 mol/L) lower than with Ca^2+^ and Sr^2+^ Crosslinking agent and the effective crosslinking degree have not significantly influenced the thermal behavior of sodium-alginate hydrogels	Compressive modulus (G) substantially higher than with Ca^2+^ and Sr^2+^ (53.8–121 kPa in deionized water and in NaCl 17.9–85.4 kPa) Effective crosslinking degree considerably higher than with Ca^2+^ and Sr^2+^ (outer, fast crosslinking obstructed ion diffusion and presented a step to a homogenous structure)
Alginate-based films containing natamycin [62]	Significant decrease in the water uptake for barium crosslinked films was observed. Ca-Ba films were more hydrophobic than Ba-Ca films	Ba^2+^ ion crosslinked films were brittle and revealed a wrinkly, whitish appearance, rougher to the touch
Alginate/polyacrylamide [63]	Swelling ratio was reduced	Stronger gel network was formed. BaFe-1/8-w (original weight ratio of sodium alginate to acrylamide of 1/8 equilibrated with water solution) showed a slight decrease in the tensile strength and the stiffness compared with BaFe-1/8-s (equilibrated with salt solution) Semi-crosslinked Ba-Alg network, due to weak interaction between Ba^2+^ and COO^−^ on the M blocks and unpaired G blocks, contributing to the weaker strength and tensile stress
Alginate-Based Microcapsules [65]	No data	Microcapsules (crosslinked with 0.5% BaCl_2_) were imperfectly spherical, mainly elliptical, moderately broken, with an irregular surface, demonstrating many exposed cells in the outer part of the structure 1% or 1.5% BaCl_2_ gelling solutions displayed significantly better morphological characteristics
